# Feasibility of a multidimensional home-based exercise programme for the elderly with structured support given by the general practitioner's surgery: Study protocol of a single arm trial preparing an RCT [ISRCTN58562962]

**DOI:** 10.1186/1471-2318-9-37

**Published:** 2009-08-17

**Authors:** Timo Hinrichs, Claudio Bucchi, Michael Brach, Stefan Wilm, Heinz G Endres, Ina Burghaus, Hans-Joachim Trampisch, Petra Platen

**Affiliations:** 1 Department of Sports Medicine and Sports Nutrition, Ruhr-University Bochum, 44780 Bochum, Germany; 2 Institute of Sport Science, University of Münster, Horstmarer Landweg 62b, 48149 Münster, Germany; 3 Institute of General Practice and Family Medicine, University Witten/Herdecke, Alfred-Herrhausen-Straße 50, 58448 Witten, Germany; 4 Department of Medical Informatics, Biometry and Epidemiology, Ruhr-University Bochum, 44780 Bochum, Germany

## Abstract

**Background:**

Physical activity programmes can help to prevent functional decline in the elderly. Until now, such programmes use to target either on healthy community-dwelling seniors or on elderly living in special residences or care institutions. Sedentary or frail people, however, are difficult to reach when they live in their own homes. The general practitioner's (GP) practice offers a unique opportunity to acquire these people for participation in activity programmes. We conceptualised a multidimensional home-based exercise programme that shall be delivered to the target group through cooperation between GPs and exercise therapists. In order to prepare a randomised controlled trial (RCT), a feasibility study is being conducted.

**Methods:**

The study is designed as a single arm interventional trial. We plan to recruit 90 patients aged 70 years and above through their GPs. The intervention lasts 12 weeks and consists of physical activity counselling, a home-exercise programme, and exercise consultations provided by an exercise therapist in the GP's practice and via telephone. The exercise programme consists of two main components: 1. a combination of home-exercises to improve strength, flexibility and balance, 2. walking for exercise to improve aerobic capacity. Primary outcome measures are: appraisal by GP, undesirable events, drop-outs, adherence. Secondary outcome measures are: effects (a. motor tests: timed-up-and-go, chair rising, grip strength, tandem stand, tandem walk, sit-and-reach; b. telephone interview: PRISCUS-Physical Activity Questionnaire, Short Form-8 Health Survey, three month recall of frequency of falls, Falls Efficacy Scale), appraisal by participant, exercise performance, focus group discussion. Data analyses will focus on: 1. decision-making concerning the conduction of a RCT, 2. estimation of the effects of the programme, detection of shortcomings and identification of subgroups with contrary results, 3. feedback to participants and to GPs.

**Conclusion:**

A new cooperation between GPs and exercise therapists to approach community-dwelling seniors and to deliver a home-exercise programme is object of research with regard to feasibility and acceptance. In case of success, an RCT should examine the effects of the programme. A future implementation within primary medical care may take advantage from the flexibility of the programme.

**Trial registration:**

Current Controlled Trials ISRCTN58562962.

## Background

Home-based exercise for the elderly has been shown to be effective in keeping and increasing functional and health status, when carried out correctly. Low price and organisational flexibility make it an alternative or a supplement to other forms of physical exercise and active lifestyle. With increasing age, the general practitioner's surgery often becomes a distinguished place of health care and social contacts. Our approach is to make use of this reputation and to set up cooperation between general practitioners and exercise therapists. Thus, elderly people should be invited to and guided through a state of the art home-based exercise programme. In order to prepare a randomised controlled trial (RCT, phase III, in accordance with [1]) on the new programme, a feasibility study (phase II) will be conducted. Provided satisfying results, the cooperation model could be implemented into every day primary medical care for the aged (phase IV). The following sections describe the background and development of the programme, and the methods and measures of the feasibility study. Criteria for the RCT are given in section "Statistical analysis and decision-making on RCT preparation".

### Physical activity and fitness in old age

In the face of demographic chance [2], preserving functional status, mobility and thus independence in old age is more and more challenging for the society. Some 80% of the population in Germany, aged 70 to 85 years, stated to suffer from two or more chronic diseases, 24% from five or more [3]. Without any question, physical activity on a regular basis contributes to healthy ageing. Positive influences both on chronic diseases such as diabetes or coronary heart disease, and on preservation of mobility and independence are well documented [4-6]. Results from Peel et al. [7] show a positive correlation between functional mobility (leg strength, balance, locomotion speed) and behavioural mobility (utilizing certain neighbourhoods and places inside and outside town). In order to obtain a standard value of life span postural balance, Era et al. [8] included 7979 Finnish subjects aged 30 years or older in a balance test battery on a force plate. Slight decreases in balance scores were already observable in middle-aged subjects (age 40 years and older) but became more pronounced after the age of 60 years. Reduced balance has been identified as a risk factor for falls and is related to decreased strength [9]. Hurley [10] reports a decrease in muscular strength of about 12–15% per decade, starting with the fourth and fifth decade (up to the eighth decade) of life. The results of Jette and Branch [11] may describe another functional consequence of this reduction: only 72% of men and 34% of women in a sample aged 75–84 years of age reported to being able to lift a weight over 10 lbs., comparable to a full shopping bag. Other aspects of physical fitness also show a clear relationship to everyday functioning: Maximum oxygen uptake, a measure of endurance capacity, decreases by 3 to 6% per decade during the third and fourth decade, and by more than 20% during the eighth decade of life [12]. Thus, the performance of some everyday activities seems to demand maximal effort from many old persons. Geraldes et al. [13] obtained a positive correlation between hip flexibility and functional test scores (sit-to-stand, going-up-stairs, 6-minute-walk) in senior women. Kinematic analyses with young and old subjects identified restriction of shoulder flexion, knee extension and ankle plantar flexion as main age related and aggravating influences on everyday movements and activities, e.g. sit-to-stand, walk, arm operations [14]. Summing up, even highly aged and chronically sick persons may profit from physical exercise of endurance, strength, balance and flexibility [15-19]. Consequently, present guidelines of the American Heart Association (AHA) and the American College of Sports Medicine (ACSM) demand for multidimensional activity programmes, which should include a daily training of these four basic motor skills [5].

### Multidimensional programmes for the elderly

In this section a review is given on multi-goal programmes targeting on endurance, strength, balance and flexibility of the elderly. The question arises, if a combination of different training goals in fact can result in significant and relevant improvements of activities of daily living and quality of life for people in old age. Baker et al. [20] went into that matter with a systematic review on randomised controlled trials (RCT) of multidimensional training interventions with people aged 60 years or older. They included 15 trials aiming at least at improvements of endurance, strength and balance; improvement of flexibility was an optional aim. The outcomes were heterogeneous between the trials. The authors could only confirm an effect on the reduction of falls; influences on functional abilities and quality of life seemed to be rather small. A possible selection bias causing the latter is discussed, because sample baseline measurements were quite well (low functional restrictions, high quality of life). Mian et al. [18] studied the effect of training interventions on locomotor function of the elderly. Inclusion criteria of this systematic review were met by 55 trials. In 36 (80%) out of 45 trials with a sedentary control group, at least one locomotor function improved in the experimental group relative to the control group. Most frequently used outcome measures were habitual walking speed (22 trials) and timed-up-and-go test (TUG, 16 trials). TUG scores improved significantly in 13 studies (by 5 to 28%). Only 10 trials (18%) were multidimensional, targeting at least on strength and endurance and balance, optionally on flexibility. Only one of them utilised home-based exercise. Another systematic review [21] focused on functional abilities of frail elderly and comprised 20 papers. These were based on a total of 23 different interventions, which included strength exercise (9), tai chi (2) or combinations of strength training with endurance or flexibility or balance training (12). Mostly, elastic tapes, dumb bells and ankle weights were used for strength exercises. Three trials utilised special strength training machines. Merely two of the interventions were purely home-based. These were monitored by physiotherapists. The other interventions consisted of group exercise, carried out in training facilities. Five of them offered combined facility and home exercise. Paw et al. [21] noticed a broad variety of functional outcome measures. A majority of the interventions (14 out of 20) resulted in effects on at least one of the functional parameters. Yamauchi et al. [22] conducted an RCT on the effects of a multidimensional 12-week home-based exercise programme, which contained walking, stretching, and resistance exercises for 23 elderly people. Once a week, a group session was offered in the community centre. After 12 weeks all functional parameters (arm curl, chair stand, TUG, back scratch, sit-and-reach, 12-minute-walk) had improved significantly in relation to the control group (n = 17).

### Home-based versus centre-based exercise

Obviously, there are some advantages of home-based exercise in opposition to group session exercise: Special facilities or costly devices are not necessary, and so is the transport – which might be most important with respect to the target group. Thus costs are low and the training can be conducted daily – just as demanded by ACSM and AHA. Under a public health perspective, the access to home-exercise programmes seems to be easier. Once implemented, a home-exercise programme should be capable to reach a broad audience. Ashworth et al. [23] conclude from their Cochrane Review, that especially on the long run, home-based exercise programmes show better adherence than centrally conducted programmes. Inauspicious or distant locations may result in low adherence to a programme [24]. On the other hand, group exercise may enjoy popularity on the strength of social contacts, which generally are of very high importance for the target group. But yet there are no interventional trials comparing home-based single training versus centre-based group training and controlling all other variables, e.g. intensity, volume, and structure of exercise [18]. Therefore, a final statement or a general decision for either organisation of exercise is impossible at the moment. In order to maximise compliance to a home-based programme, exercises should be simple, combined with proper equipment and personal support: Henry et al. [25] found higher compliance for participation in a programme with two exercises compared to programmes with five or eight exercises. Schoo et al. [26] failed to improve compliance or exercise performance of patients with osteoarthritis of the knee, when they handed them audio or video cassettes in addition to an oral instruction and a written exercise booklet for their home exercises. Compared with that, stakeholders in home-based exercise emphasise the importance of personal contact, given by oral instructions and phone support [27,28].

### Approaching seniors at home and via general practitioners

Until now, activity programmes used to target on healthy, community-dwelling seniors or on elderly living in special residences or care institutions [[29], for an example]. Sedentary or frail people living in their own homes are difficult to reach. About one half of seniors aged 85 years or above rarely leave their homes [30,31]. Interventional trials targeting on those "housebound" aged are scarce. Therefore, the surgery of the general practitioner (GP) seems to be an ideal starting point and junction for this target group: (a) the physician knows best about the eligibility of the individual, (b) there are chances to recruit patients for the programme and guide them through it, and (c) extra home visits could help to increase adherence. These aspects are discussed in the following sections.

#### Assessment of exercise eligibility

Especially in old age, the question of eligibility for some exercise arises. According to Cress and colleagues [32], a prior medical examination is not required for asymptomatic seniors taking up light exercise. But examination is recommended before taking up moderate or high intensity exercise, because the presence of one or more chronic diseases is quite normal in old age. The GP knows his or her patient, often for many years, and is able to judge about his or her health status. In addition, the examination and judgement by the physician seems to be more valid than specialised test instruments: With respect to the large benefit of physical activity and the relatively low risk of serious cardiovascular incidents, current guidelines of the American Heart Association, of the American College of Cardiology [33,34], and the U.S. Preventive Services Task Force [35] no longer dissuade from taking up light or moderate exercise without previous exercise tolerance testing. Only rarely acute cardiovascular incidents happen as a result of physical activity. Furthermore, they are not predictable: Neither screening questionnaires like rPAR-Q [36] nor exercise electrocardiograms (ECG) are valid diagnostics to identify individuals, who are in danger of such incidents [37,38]. Both sorts of instruments yield high rates of false positives and false negatives, e.g. the sensitivity of exercise ECG for future cardiovascular incidents (3–12 years) is 40–62% [35,37].

#### Acquisition of participants

Generally, the trustful relation of patient and doctor plays an important role in patient compliance [39,40]. Therefore, GPs have chances to help their patients change their activity habits, and in particular to acquire them for participation in physical exercise programmes. Elley et al. [41] conducted a controlled trial with physicians advising their patients. This resulted in an increased physical activity and health-related quality of life. Unfortunately, only few primary care physicians spend time on motivating their patients to reach for more physical activity or on discussing its benefits [41,42]. This might be due to lack of time or to lack of specialised knowledge. A well-structured programme could yield support for both aspects.

#### Home visits

Home visits by trained personnel are one possible way to reach sedentary or frail elderly people in their homes. Tudor-Locke et al. [43] delivered an exercise intervention through existing home support infrastructures for old people. Home support workers instructed their clients on how they could exercise and provided ongoing encouragement on occasion of regular home visits. McMurdo and Johnstone [44] recruited participants for an interventional trial in sheltered housing complexes. For a period of 6 months, a physiotherapist visited them at home every 3 or 4 weeks and instructed them in home-based exercises. In a study of Campbell et al. [45], 622 potential participants were chosen from patient card files of 17 GPs surgeries and invited by the physicians. 233 could be randomised. The intervention consisted of four home visits within 2 months by a physiotherapist with instruction in a 30 minute three times a week exercise programme, and also in three walks a week. Following, there were regular phone calls over a period of 10 months, to stay in contact and to keep adherence.

### Summary and consequences for programme development and trial methodology

Present guidelines for physical activity demand multidimensional programmes. It is a matter of research, how such programmes may in fact improve clinically relevant parameters, everyday functions or quality of life for elderly people. So far, interventions used to target independent seniors or residents of care institutions. There are problems to recruit community-dwelling, but sedentary aged with restricted mobility. Approaching them via their GP seems promising. In this case, the physician plays a decisive role both in acquisition and assessment of potential participants. A well-designed programme utilising this "surgery approach" should support the physician with structured information and personnel. An exercise therapist can provide this kind of support: he or she is able to take individual needs and constraints, but also principles of modern training into account [46]. Especially in older adults with multiple morbidities, the development of a tailored and safe exercise programme requires knowledge, time, individualised mentoring and a close cooperation between GP and exercise therapist. Considering health-related heterogeneity and restricted mobility, group exercise in central facilities seems suboptimal compared to home-based exercise. In summary, a home-based exercise programme, approaching and supporting community-dwelling but mobility-restricted seniors via their GP and an exercise therapist, is object of research with regard to feasibility and acceptance. In case of success, a randomised controlled trial should examine the effects of the programme. After positive evaluation, a future implementation within primary medical care may take advantage from the flexibility and individuality of the programme.

### Development of the exercise programme

Cross-sectional and interventional studies have proven that functional abilities can be increased by physical training even in very old people and in elderly patients with chronic diseases [15-19]. According to the current ACSM/AHA recommendations on physical activity in older adults [5], a multidimensional exercise programme has been designed, that: 1. aims to improve aerobic capacity, strength, flexibility and balance; 2. can be individually adapted to the participant's functional abilities; and 3. allows combining preventive and therapeutic aspects of exercise. An activity plan, an activity log and a pedometer will be used to improve motivation for and adherence to the exercise programme. An overview of the home-based exercises is given in table 1.

**Table 1:** Overview of home-based exercises.

Strengthening Exercises		
15 dynamic repetitions per exercise 3 sets		
	
	Basic Exercise	Intensive Exercise
	
1.	Seated (on a chair) knee extension (leg curl)	Seated (on a chair) knee extension (leg press) against elastic resistance
2.	Standing leg abduction (holding onto a chair)	Standing leg abduction against elastic resistance (holding onto a chair)
3.	Standing calf raise, two-legged (holding onto a chair)	Standing calf raise, single-legged (holding onto a chair)
4.	Standing press-up against the wall	Standing pectoral press (palms of hands against each other) with a 3-second isometric phase
5.	Seated sit-ups	Lying sit-ups
6.	Seated upper back (retraction of scapulae) against elastic resistance	Standing upper back (retraction of scapulae) against elastic resistance with a 3-second isometric phase
7.	Shoulder adduction against elastic resistance	Shoulder adduction against elastic resistance with a 3-second isometric phase
8.	Triceps curl against elastic resistance	Triceps curl against elastic resistance with a 3-second isometric phase
9.	Biceps curl against elastic resistance	Biceps curl against elastic resistance with a 3-second isometric phase
10.	Shoulder abduction against elastic resistance	Shoulder flexion against elastic resistance
**Flexibility Exercises (Static Stretching)**		
holding each position for 15 seconds 3 repetitions		
	
	Exercise (intensity varies with position)	
	
11.	Seated sit-and-reach	
12.	Seated quadriceps stretch	
13.	Standing pectoralis stretch against the wall (flexed elbow)	
14.	Standing upper back stretch (protraction of scapulae)	

**Balance exercises**		
holding each position for 15 seconds 3 repetitions		
	
	Basic Exercise	Intensive Exercise
	
15.	One-leg-stand (slightly holding onto a chair)	One-leg-stand (slightly holding onto a chair) with the other leg raised to the front with extended knee
16.	Tandem stand (slightly holding onto a chair)	Tandem walk (10 steps, slightly holding onto a chair or table)

The first column gives the order of the exercises. The second and, if present, the third column contain descriptions of two levels of intensity

#### Strengthening exercises

In order to train all major muscle groups, strengthening exercises comprise a combination of three lower body exercises, one trunk exercise and six upper body exercises. Participants are instructed to perform a 5 to 10 minute warm up (walking in place) before starting with the exercises. To ensure the possibility of adapting the exercise to the participant's performance level, every exercise can be performed in two different variations: less intensive ("basic") or more intensive ("intensive"). E.g., the exercise to strengthen the calf muscles (standing calf raise) can be performed two-legged ("basic") or single-legged ("intensive"). Some exercises are to be performed using an elastic resistance band. Elastic resistance band exercises have been demonstrated to be efficient in improving physical function in independently-living older adults [47] and in physically frail community-dwelling older persons [48]. Elastic resistance bands offer another possibility to adapt the level of difficulty to the participant's performance level as they are available in different colour-coded levels of resistance. Depending on the performance and the progress, participants can be instructed to increase intensity by advancing to the next resistance colour of the elastic band. The level of effort should be moderate (5 or 6 on a 10-point scale, where no movement is 0, and maximal effort of a muscle group is 10). Participants should perform 3 sets of 15 repetitions of every exercise. They are instructed to not hold their breath during the exercises in order to prevent exercise induced blood pressure elevations.

#### Flexibility exercises

To maintain (or improve) the range of motion necessary for daily activities, the programme contains two upper body and two lower body flexibility exercises (static stretching). Participants are instructed to perform the exercises slowly, holding each position for 15 seconds. They are instructed to stretch to a point of moderate tension without pain in the joints or muscles, gradually increasing the range of motion. The flexibility exercises should be performed after the strengthening exercises.

#### Balance exercises

To reduce risk of injury from falls and to improve mobility [9], the programme comprises two balance exercises that can be performed in two different variations ("basic" or "intensive") depending on the participant's abilities. To perform these exercises safely it is important that participants follow the safety instructions given by the exercise therapist (e.g., tandem walk along a stable table).

#### Walking

Home-based walking has been shown to be effective in improving cardiopulmonary function in community-dwelling elderly people [49]. To improve aerobic capacity, this study's participants are instructed to walk for exercise (around their homes) four times a week for 30 minutes. The level of effort should be moderate (5 or 6 on a 10-point scale, where sitting is 0 and all-out effort is 10). Depending on the physical activity habits and the abilities of the participant, it can be necessary to start with shorter walks of lower intensity. With the progress of the intervention, duration of the walks and intensity level can be increased in a stepwise manner.

### Aims of the trial

From a methodological point of view, effects should be studied by randomised controlled trials. Before spending large resources for an RCT, the feasibility of the programme should be tested, and there should be some probability that there will be an effect. Thus, the aims of the present study are:

1. On the individual level: to test the feasibility of the new physical activity programme according to the ACSM guidelines. The exercises were conceptualised and described in the previous subsection "Development of the exercise programme".

2. On the institutional level: to initiate and evaluate a cooperation between GPs and exercise specialists, for recruiting and supporting participants. The reasons for this procedure were given in the subsections "Approaching seniors at home and via general practitioners" and "Summary and consequences for programme development and trial methodology".

3. On the methodological level: to analyse the capability and functional effects of the programme including the special support scheme, and the adherence of the participants, thus gaining preliminary data for the preparation of a randomised controlled study. The decision criteria are laid down in subsection "Statistical analysis and decision-making on RCT preparation".

## Methods

### Study overview: settings, targets, design and registration

**Figure 1:**
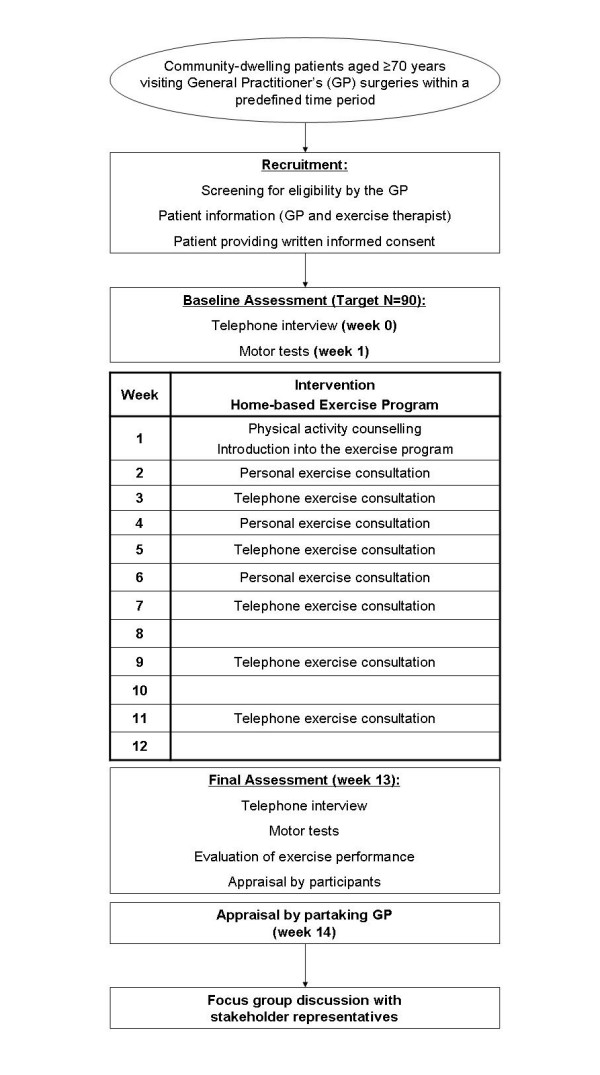
**Time table and flow chart of the trial**. The time table refers to the perspective of the participating subject. The procedure on the institutional level is described in subsection "Recruitment of subjects and time schedule".

The trial targets on community-dwelling patients, aged 70 years or above, who visit their GP's surgery. Beneath the practices, the participants' homes are also settings of the intervention, where the exercises are conducted and data are collected (diaries). For a time table and flow chart see figure 1 in subsection "Interventions". Using the framework of Campbell et al. [1], the present study belongs to phase II and is to prepare a phase III study. The trial is designed as a single arm interventional trial and is registered at http://www.controlled-trials.com as ISRCTN58562962.

### Recruitment of subjects and time schedule

Community-dwelling older adults will be recruited through GP surgeries. The recruitment period starts in April 2009 and ends in July 2009. The last participant is expected to finish the intervention in November 2009. In order to recruit a representative sample, all participants are recruited consecutively through (approximately 10) GP practices (motivated research practices associated to the Institute of General Practice and Family Medicine, University Witten/Herdecke) within predetermined time periods. All patients aged 70 years or above who are seen by the GP within his consultation hours (personal contact) within that time period are screened for eligibility. Exclusion for medical or other reasons (see exclusion criteria) is documented. If the physician considers a patient eligible for the study, the patient is informed about the study in detail by the physician and by an exercise therapist. The therapist spends the whole recruiting period in the practice to assist the GP and to document the recruitment process. After a participant has given written informed consent, appointments for the baseline assessment (telephone interview and motor tests in the practice, see secondary outcome measures) are made. The recruiting period for the respective physician ends with the recruitment of a predetermined number of patients. The recruitment period for the first participating practice ends with the recruitment of 12 patients. The number of participants to be recruited by the following practices depends on the number of patients that have already passed the baseline assessment in the previous practices. The target of this recruitment mode is that 90 subjects participate in the baseline assessment (on average 9 patients per practice if 10 practices participate in the study).

#### Inclusion criteria

To be eligible for this study, patients have to be aged greater than or equal to 70 years. All participants need medical clearance from their GP to participate in the study. They have to be able to walk (with or without a walking aid) and to visit the practice of the GP for repeated consultations. Furthermore, participants have to be able to cooperate appropriately and to follow the instructions of the home-based exercise programme (according to their GP's judgement). Finally, all participants have to provide written informed consent.

#### Exclusion criteria

Patients are excluded if the intervention doesn't fit into their schedule (e.g. because of a planned vacation). Patients are excluded for medical reasons if they suffer from untreated arterial hypertension or arterial hypertension despite antihypertensive medication (GP's judgement), from chronic heart failure (New York Heart Association (NYHA) grade III–IV), from an acute psychiatric disorder (e.g. depression), or from a severe consuming illness. Furthermore, patients are excluded, if they suffered a clinically relevant cardiovascular event (e.g. unstable angina pectoris, myocardial infarction, coronary angiography and/or angioplasty, cardiac surgery), a clinically relevant cerebrovascular event (e.g. stroke, recurrent TIA), any inpatient surgical procedure, or a deterioration of an insufficiently controlled diabetes mellitus (according to their GP's judgement) within the past 3 months, or if their HbA1c exceeds 10% (if available). Concurrent participation in another clinical trial is also an exclusion criterion.

#### Risk-benefit considerations

Unlike some physical activity trials that often exclude chronically diseased individuals this study only excludes individuals that are at high risk for adverse events. The GP, who knows the participant's medical history and his current health status, decides whether the patient is eligible for the programme. For the patients who are eligible for the study, the expected cardiovascular benefit of the programme exceeds the risk for adverse cardiovascular events. Cardiovascular risk is not the only risk associated with starting an exercise programme. An increase of physical activity and especially of walking outdoors may initially increase the risk of minor and major musculoskeletal injuries (including sprains, strains, joint pain, overuse injuries, falls and fractures) compared to a sedentary lifestyle. But in the long term, an increase in physical activity is considered to lead to a better mobility, to an improvement in gait performance and consecutively to a reduction of fall and injury risk. To reduce the risk of adverse events, the exercise programme is tailored to the abilities of the patient by an exercise therapist (in cooperation with the GP) who holds a university degree in sports science (bachelor or higher).

### Interventions

The intervention lasts 12 weeks and consists of physical activity counselling, the previously described home-based exercise programme, and exercise consultations provided by an exercise therapist in the GP's practice and via telephone. The exercise programme starts immediately after the baseline assessment in the GP's practice (week 1) and ends with the final assessment in the practice (week 13, see figure 1).

#### Physical Activity Counselling and Exercise Consultation

The exercise therapist provides initial counselling on general aspects of physical activity (week 1), an introduction (theoretically and practically) into the exercise programme (week 1), a given number of additional personal consultations within the practice of the participant's GP (week 2, 4 and 6), and a given number of consultations on the telephone (week 3, 5, 7, 9 and 11). First of all, the participant receives standardised counselling by the exercise therapist pertaining to the benefits of physical activity (week 1). The conversation is aiming to improve knowledge and understanding of the preventive and therapeutic effects of physical activity on particular chronic conditions. The counselling also contains a systematic assessment of the actual physical activity status, a discussion of potential barriers, problem solving to overcome barriers (e.g. by creating an activity timetable), and the creation of individual physical activity goals. Secondly, based on the assessment of the actual physical activity habits and the functional abilities of the participant and based on the knowledge of the participant's chronic diseases (communicated by the GP), the exercise therapist adapts the exercise programme to the participant's abilities and needs, integrating preventive and therapeutic aspects. Every participant is taught (theoretically and practically) to correctly perform the selected exercises (week 1). A pictorial guidebook is provided to each participant to assist them in correct exercise performance. Within the following personal consultations (week 2, 4, 6) with the exercise therapist, the participant gets the opportunity to discuss problems concerning the exercise programme. Exercises are controlled for correctness of performance. New exercises are taught. Choice and intensity of exercises is adapted to the participant's performance level. The consultations aim to provide encouragement and to improve knowledge and self-efficacy of the participants so that they are able to perform their activity programme independently. Support is reduced in a stepwise manner and personal consultations are replaced by telephone consultations (week 3, 5, 7, 9 and 11). If a patient misses a personal consultation in the practice, the appointment is replaced by a telephone consultation. Every consultation follows a standardised protocol and is documented by the exercise therapist.

#### Home-based exercise programme

The exercise programme (described in section "Development of the exercise programme") consists of two main components that should be performed in alternating order:

1. a combination of home-exercises to improve strength, flexibility and balance on 3 days of the week,

2. walking to improve aerobic capacity on 4 days of the week.

Every participant is instructed and supervised by his personal exercise therapist. The exercise programme usually starts with a selection of a few simple home-exercises and with short walks. Additional exercises are taught by and by (depending on the participant's progress) with the advancement of the intervention. The duration of the walks is also increased in a stepwise manner. Days of rest are accepted according to the patient's needs. In case a participant is able to perform all home-exercises, he performs ten strengthening exercises (moderate intensity 5–6/10), followed by four flexibility exercises and two balance exercises. The exercises are numbered and should always be performed in the same chronological order. To perform all home-exercises in a row it takes about 30 to 45 minutes. In case of questions or problems concerning the exercise programme, patients have the opportunity to contact their GP or their exercise therapist.

#### Materials

##### Pictorial guidebook

The pictorial guidebook includes a statement of the activity goals and general information on the exercise programme (frequency, duration, intensity), instructions for use of the pedometer and the elastic resistance bands, safety instructions, instructions for clothing and warm-up, a detailed description of all home-exercises, and contact information of the exercise therapist (including the mobile phone number). The pictorial guidebook has been developed using older adults as models.

##### Workbook

Every participant receives the German Olympic Sports Federation workbook "Bewegungsangebote 70 plus" [Physical activity choices 70 plus] [50] that includes general information on the health benefits of physical activity and detailed information about physical activity programmes for seniors in Germany.

##### Elastic resistance bands

Elastic resistance bands are provided to the participants in order to perform some of the home-exercises. The elastic resistance bands (Thera-Band^® ^GmbH, Dornburg-Frickhofen, Germany) are available in eight different colour-coded levels of resistance. Two different levels of resistance, that are rather low within the available spectrum, have been chosen for this study: the yellow band ("thin") provides lower resistance; the red band ("medium") provides higher resistance. If necessary, additional resistance can be created by folding the band. Every participant gets a yellow and a red band, each of them measuring two metres.

##### Pedometer

As a motivational support and to receive immediate feedback on their accumulated step count, every participant gets a pedometer (NL-800, New-Lifestyles Inc., Lee's Summit, USA). The pedometer measures vertical acceleration to count steps. It has an internal memory chip and is capable of storing up to 7 days of data, in 1-d epochs. The internal clock resets the step count at midnight. Step total is displayed on a screen. The pedometer should be worn close to the hip, attached to a belt, waistband or a horizontal pocket. Participants are instructed in the use of the pedometer and asked to begin recording daily pedometer counts (every evening).

##### Activity Log

Patients are instructed to record their daily activity. The activity log serves as a motivational support and as a help for the therapist to supervise (and adapt) the exercise programme. The following items are recorded by the patients every day: duration of walking for exercise, performance of home-exercises, total step count, comments (e.g. reasons for inactivity). The activity log also serves as an outcome measure.

##### Consultation protocol

The exercise therapist completes a structured protocol for every consultation (personal/telephone). The protocol serves as a documentation of the participant's progression and as a help for the therapist to pick up on the last consultation and to further develop the participant's exercise programme. The following items of the protocol are based on a structured dialogue between the therapist and the participant: general well-being of the participant, activity log use (problems, documentation of exercises and steps, inactivity), pedometer use (problems, usage, frequency and duration of use), walking for exercise (problems, frequency, intensity, duration), home-exercises (problems, chosen exercises, frequency, intensity, duration), medical consultations (reason, specialisation of the consultant) or hospital admissions (reason, hospital department). During the personal consultations in the GP's practice, the structured dialogue is followed by an assessment of the correctness of exercise performance (correction, adaptation of resistance, progression from "basic" to "intensive" exercises) and by rehearsing new exercises. At the end of the consultation, the patient gets an adapted exercise programme (duration and intensity of walks, selection of home exercises), that is documented in the consultation protocol and in the participant's activity log. The consultation protocol may be analysed and used as an outcome measure in case of drop-outs.

### Outcome measures, types of data and measurement techniques

We discriminate primary measures, which are essential to judge on the cancellation or conduction of the planned RCT, from secondary measures, which are all other data to be obtained.

#### Primary outcome measures

##### Appraisal by participating general practitioners

A structured interview will be held with partaking GPs. The main questions are: Would you once again participate in the trial? Why/why not? Do you have any ideas or proposals for programme improvements? In addition, age and sex of the physician and profile data of his or her practice are documented (urban or rural area, medical field, single handed practitioner or group practice, quarterly number of patients seen below 900, between 900 and 1500, above 1500).

##### Documentation of any undesirable event during the intervention

Any symptom or disease of any participant, which occurs during the intervention, is called an undesirable event. This definition is valid without reference to the fact, whether the intervention might be a cause or not. In case of an undesirable event, the participant promptly has to suspend exercise and to visit the surgery. The event has to be documented by a standardised report protocol, including the following judgements by the physician: medical/non-medical; caused/not caused by exercise; subject may/may not continue exercises.

##### Measures of drop-out

For each case of discontinued participation, the following reasons will be discriminated: medical reason (*r*_1_), change or misjudgement of inclusion or exclusion criteria (*r*_2_), subject's own decision (*r*_3_). After that, certain sets (groups) will be aggregated:

*A *– all participants who passed the baseline assessment

*DO*_*i *_– participants who drop out by reason *r*_*i*_

*DO *= *DO*_1 _∪ *DO*_2 _∪ *DO*_3 _– all participants who drop out

A* = *A*\(*DO*_1 _∪ *DO*_2_) – adjusted set of participants (all participants not constrained to abort exercise by medical or trial criteria reasons)

DO* = A* ∩ *DO*_3 _– adjusted drop out group (participants who discontinue on their own decision). Members of this group will be asked for a personal reason (voluntary and open question), and also be asked to participate in the final assessment.

The following parameters are of special interest:

|*DO*| – total number of participants who drop out after the initial assessment

|*DO*_*i*_| – number of participants who discontinue for reason *R*_*i *_

|DO*|/|A*| – adjusted drop out rate

##### Measures of adherence

For the adjusted set of participants (A*), measures of adherence are calculated:

• Number of consultation dates with the exercise therapist attended (personal and telephone exercise consultations)

• Number of activity units (walks, home exercises) documented in the activity log. Depending on the data available, corresponding numbers will also be calculated for DO*.

#### Secondary outcome measures

##### Effect measures (week 0/1 and week 13)

To measure effects of the intervention on functional skills, every day physical activity, general health, frequency of falls and fear of falling, the following measures will be used:

a. Motor tests (in standardised order): timed up and go, chair rising, grip strength, tandem stand, tandem walk, sit-and-reach;

b. Telephone interview: PRISCUS-Physical Activity Questionnaire (PRISCUS-PAQ), Short Form 8 Health Survey (SF-8™ 4-week recall version), three month recall of frequency of falls, Falls Efficacy Scale – International Version (FES-I).

The "timed up and go" (TUG) test has been published in 1991 by Podsiadlo and Richardson [51] as a modified version of the "get up and go" test originally published by Mathias et al. [52]. The patient is timed while he rises from an arm chair, walks 3 meters, turns, walks back, and sits down again. The test is used to quantify mobility and coordination and has been demonstrated to be a sensitive and specific measure for identifying older adults who are prone to falls [53]. The "chair rising" test, a timed test of five repetitions of rising from a chair (arms folded across the chest) and sitting down, is used to measure lower body strength [54]. Grip strength of both hands is estimated using a vigorimeter [55]. The vigorimeter (Martin GmbH, Tuttlingen, Germany) is a pneumatic squeeze dynamometer that measures the pressure the hand is able to apply around a rubber ball (three attempts per hand). Adaptation to the individual hand anthropometry will be assured by using two different ball sizes. For test of standing balance, the subjects are asked to attempt to maintain their feet in the tandem position (heel of one foot directly in front of the other foot) for 10 seconds [56]. Three attempts are timed. For test of walking balance, the subjects are asked to attempt to walk heel of one foot directly in front of the other foot (tandem walk) for 8 steps. The number of performed steps is counted and documented (three attempts). A chair sit-and-reach test is used as a measure of hamstring flexibility [57]. The PRISCUS-PAQ has been developed to measure physical activity of the past 7 days in older adults (aged equal to or greater than 70 years) by telephone interview. General health will be assessed by the use of the Short Form 8 Health Survey (SF-8 4-week recall version). This instrument yields an 8-part profile of functional health and well-being. Two SF-8 composites can be calculated: the SF-8 physical component score (PCS) and the SF-8 mental component score (MCS). These scores are linear combinations of the eight items based on their respective importance for physical functioning and mental functioning. Higher scores represent better health status. The survey has demonstrated good reliability and validity [58]. Frequency of falls (three month recall) and fear of falling (Falls Efficacy Scale-International Version (FES-I) [59], will also be assessed by telephone interview.

##### Appraisal by participant (week 13)

Participants who completed the intervention will be asked to rate the utility of the following programme actions and devices (on a scale from 1 to 6 modified from [27]: introduction into the exercise programme, personal and telephone exercise consultations, pictorial guidebook, activity log, pedometer and elastic resistance bands. Participants will also be asked to rate the length of the programme, the frequency of phone and personal exercise consultations, and the recommendation for extent and intensity of exercise. Finally, an open question on concrete proposals for programme improvements will be asked.

##### Evaluation of exercise performance by the exercise therapist (week 13)

To identify exercises that are more and exercises that are less suitable for a home-based exercise programme, the performance of the patients will be assessed at the end of the programme in terms of whether they are doing their exercises correctly. All participants who completed the intervention will be rated by their exercise therapist for correctness of performance of all exercises that have been practised within the course of the programme. A three grade scale [60] is used: grade 1 indicates that the exercise is done so well that the goal of treatment is reached; grade 2 indicates that the exercise is not carried out correctly and the goal is not reached, but that no negative impact is to be expected; grade 3 indicates that the exercise is performed incorrectly and the goal is not reached and that there is reason to believe that the exercise might have harmful effects.

##### Focus group discussion with stakeholder representatives

As an additional qualitative measure, a focus group discussion with stakeholder representatives (investigators, exercise therapists, GPs, practice team, participants, drop outs) will take place at the end of the study.

### Statistical analysis and decision-making on RCT preparation

The main purposes for data analyses are the decision on a subsequent trial, the improvement of the programme and proper feedback to the participating individuals and physicians.

#### Criteria to decide on preparation of an RCT

In order to decide for or against the conduction of a randomised controlled trial on the new programme, three criteria based on primary outcome measures (see previous subsection) were set up. If one of them is met, the plans for an RCT will be cancelled:

**Criterion 1**. In the subsequent appraisal interview, more than 50% of participating GPs answer "no", when asked if they once again would participate in the trial, and the reasons for this answer seem unchangeable.

**Criterion 2**. More than 30% of the participants have to stop exercises for medical reasons, which the physician judges to be probably caused by the intervention.

**Criterion 3**. The adjusted drop out rate (see section "primary outcome measures – measures of drop-out") is above 60%, and no simple change of the criteria would clearly level down the adjusted drop out rate.

These three requirements will be analysed using binomial tests.

#### Data exploration

Additionally, explorative data analysis will be performed to estimate the effects of the programme, to detect shortcomings and perhaps to identify certain subgroups with contrary results or special problems.

#### Individual feedback

Each participant will receive a structured feedback on his or her personal results. GPs will be handed out aggregated results.

#### Sample size

There are 3 criteria to decide if an RCT should be conducted. The power calculation is based on criterion 3, that means the adjusted drop out rate should be less than 60% to conduct an RCT. We expect more than half of the patients to actually complete the exercise programme and therefore assume a drop out rate of 45%. Given these assumptions a sample size simulation for a binomial test results in 90 patients (power = 80%, alpha = 5%).

### Ethical approval

The research carried out will be in compliance with the Helsinki Declaration. The protocol has been approved by the Ruhr-University of Bochum (Germany) Ethics Committee on 10 February 2009 (Reg.-No. 3054-07).

## Discussion

We conceptualised an activity programme for community-dwelling seniors, which

• on the individual level, contains multidimensional exercises according to accepted guidelines, and

• on the institutional level, establishes a cooperation between general practitioners and exercise therapists.

From the methodological point of view, clear criteria for the decision on the next step of development are given: A randomised controlled trial as phase III makes sense, if and only if phase II level of development was successful. Otherwise, the programme has to be changed fundamentally and phase II has to be passed through again. While the training effectiveness and patient eligibility are not expected to turn out as problems, another aspect may be crucial: The adherence of initially motivated participants. Both the design of the programme and the trial emphasise this aspect. The authors also were assured by a study of Armit and coworkers [61], which appeared after planning the trial: Inactive patients (50–70 years of age) from two GP practices were randomised into three groups, for behaviour change advice by the physician, or by the physician in collaboration with an exercise scientist. The third group received collaborative advice and a pedometer. Self-reported physical activity time increased for all groups, but the most intense intervention (collaborative advice and pedometer) yielded the highest probability for the patients to meet physical activity guidelines. In summary, a home-based exercise programme, approaching and supporting community-dwelling but mobility-restricted seniors via their GP ("surgery approach") and an exercise therapist, is object of research with regard to feasibility and acceptance. In case of success, a randomised controlled trial should examine the effects of the programme. After positive evaluation, a future implementation within primary medical care may take advantage from the flexibility and individuality of the programme.

## Competing interests

The authors declare that they have no competing interests.

## Authors' contributions

PP, HJT and TH initiated the study and raised the research grant. TH and CB developed the multidimensional home-based exercise programme. All authors substantially contributed to conception and design of the study by giving relevant intellectual input on all aspects of the study within several detailed discussions and meetings. SW recruited the participating GPs. All authors contributed to the preparation of the manuscript. TH and MB finally wrote the manuscript. All authors revised the manuscript critically for important intellectual content. All authors approved the version to be published.

## Pre-publication history

The pre-publication history for this paper can be accessed here:

http://www.biomedcentral.com/1471-2318/9/37/prepub
